# Bilateral Pronation Type Galeazzi Fracture Dislocation

**DOI:** 10.7759/cureus.64552

**Published:** 2024-07-15

**Authors:** Abdullah Zaher, Imad Marzak, Noureddine Sekkach

**Affiliations:** 1 Orthopedics and Traumatology, Delafontaine Hospital, Saint-Denis, FRA

**Keywords:** galeazzi, wrist trauma, open reduction internal fixation, druj instability, galeazzi fracture-dislocation

## Abstract

The Galeazzi fracture dislocation, an uncommon forearm fracture in adults, becomes even rarer when occurring bilaterally, with limited studies documenting this condition. Here, we report the case of a 57-year-old female who presented to our emergency room with bilateral Galeazzi fracture dislocations. The patient underwent bilateral open reduction and internal fixation of the radial fractures, along with stabilization of the distal radioulnar joints. Follow-up monitoring over 16 months postoperatively showed positive outcomes without complications.

## Introduction

The Galeazzi fracture dislocation involves a distal third radial shaft fracture accompanied by a distal radioulnar joint (DRUJ) injury. This fracture type comprises less than 10% of adult forearm fractures. Consequently, bilateral Galeazzi fractures are exceptionally uncommon, with scant literature available that describes such cases [1-6].

The mechanisms of injury typically involve either direct dorsolateral wrist trauma or, more commonly, a fall onto an outstretched hand with the wrist in pronation or supination [7]. Diagnosis is often suspected upon observing radioulnar joint widening on AP wrist radiographs and volar/dorsal subluxation on lateral wrist radiographs.

As described by Campbell, this injury is termed a “fracture of necessity” due to its complex nature, necessitating surgical treatment for optimal functional outcomes in adults [8,9]. The surgical approach typically involves open reduction and internal fixation (ORIF) of the distal radius, followed by evaluation of DRUJ stability. This assessment may lead to immobilization through pinning or, in some cases, additional ORIF procedures.

The following case report details the management of bilateral Galeazzi fracture dislocations in a 57-year-old female who underwent surgical treatment and was monitored for 16 months to assess outcomes.

## Case presentation

We present the case of a 57-year-old woman with a past medical history of hypertension who presented to our emergency department with the chief complaint of bilateral wrist pain. The history dated back a few minutes before the presentation, when she had tripped on the sidewalk while running and sustained a fall onto her two outstretched hands.

**Figure 1:**
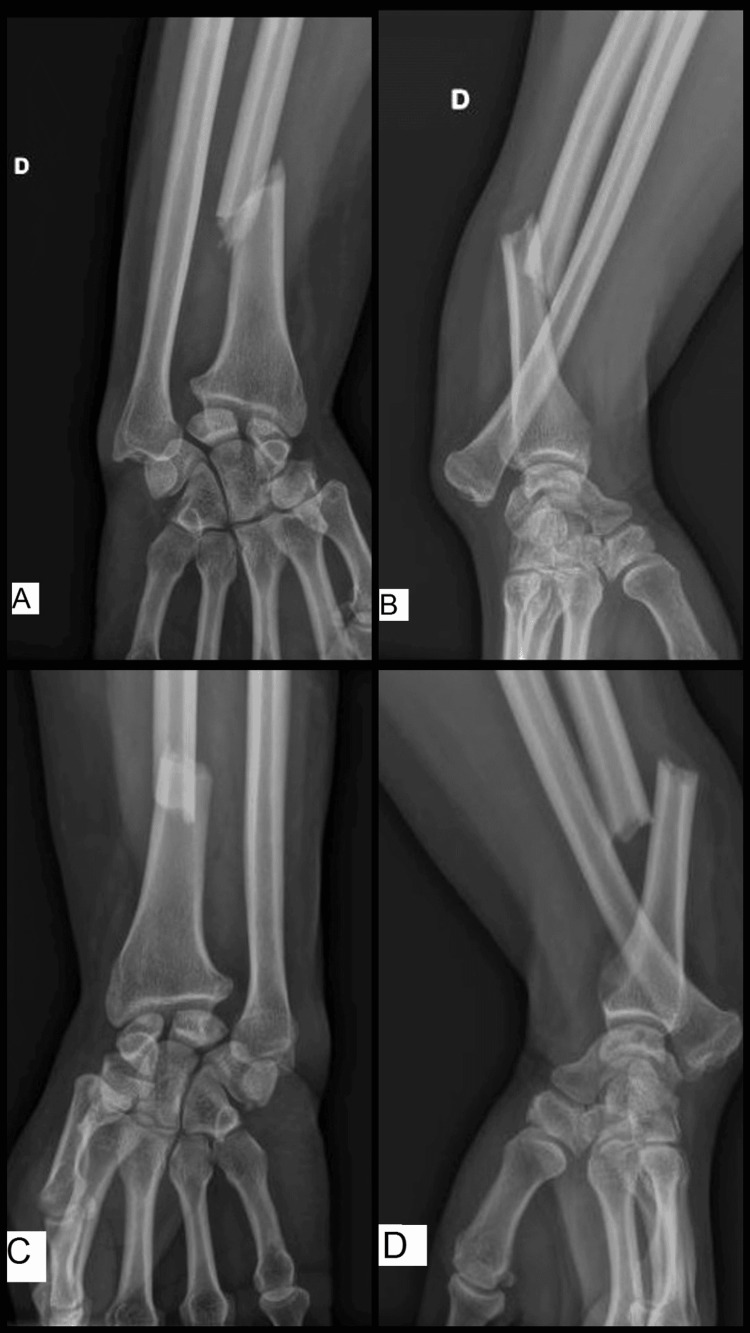
Preoperative X-rays Emergency room X-rays of the right wrist, including AP (A) and lateral (B) views, and X-rays of the left wrist, including AP (C) and lateral (D) views, demonstrating bilateral pronation type Galeazzi fracture dislocations.

At presentation, the patient was in severe pain with bilaterally deformed distal forearms and prominent bones, yet without any wound or skin opening. The neurovascular exam was reassuring. X-rays of both forearms revealed bilateral pronation type Galeazzi fracture dislocations (Figure 1). Both forearms were temporarily splinted, and the patient was hospitalized before being transferred to the operating room the next morning.

In the operating room, under general anesthesia and antibiotic prophylaxis, we began with the right side, using a tourniquet set at 250 mmHg for 53 minutes.

**Figure 2:**
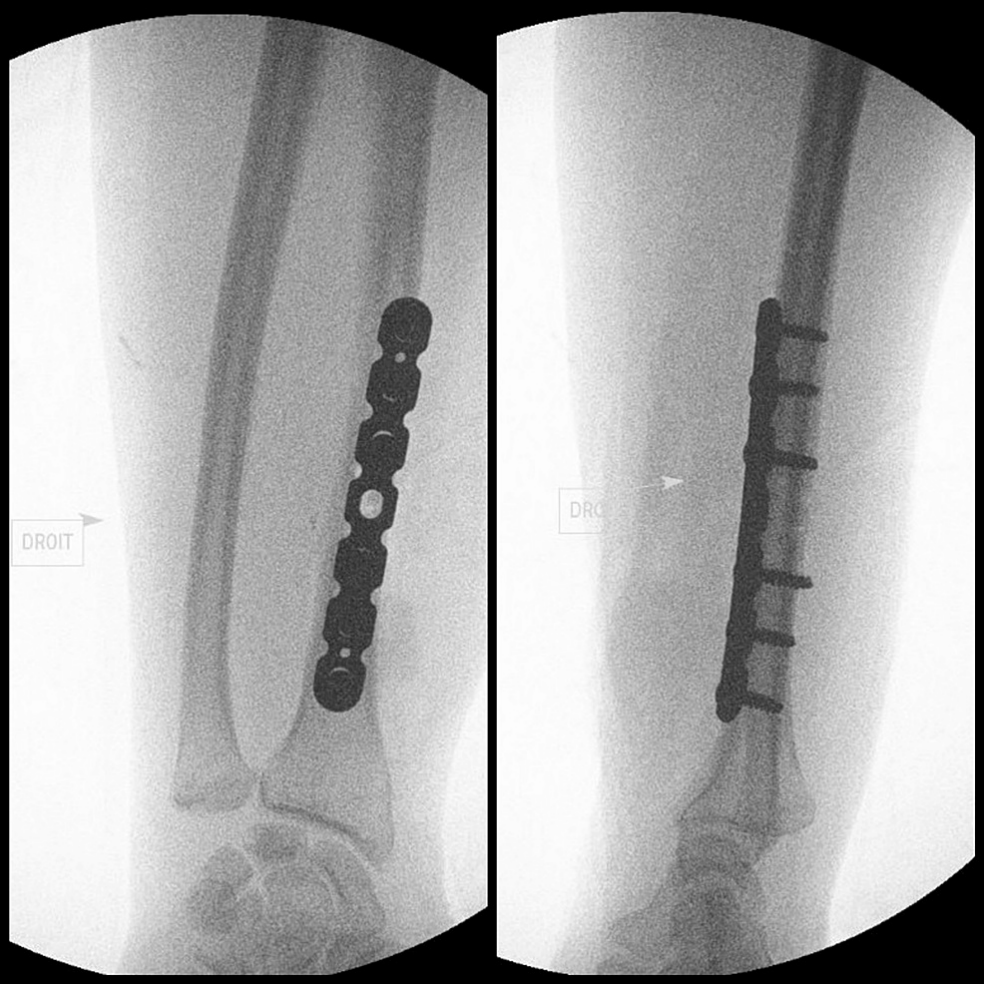
Intraoperative fluoroscopy Fluoroscopic images in the operating room showing AP (left) and lateral (right) views of the right wrist following the fixation of the radius fracture.

Using a standard Henry’s anterior approach, the fracture site was exposed. The reduction posed no particular difficulties, as reduction forceps maintained it before a seven-hole compression plate was placed and secured by three screws on either side of the fracture site (Figure 2).

**Figure 3:**
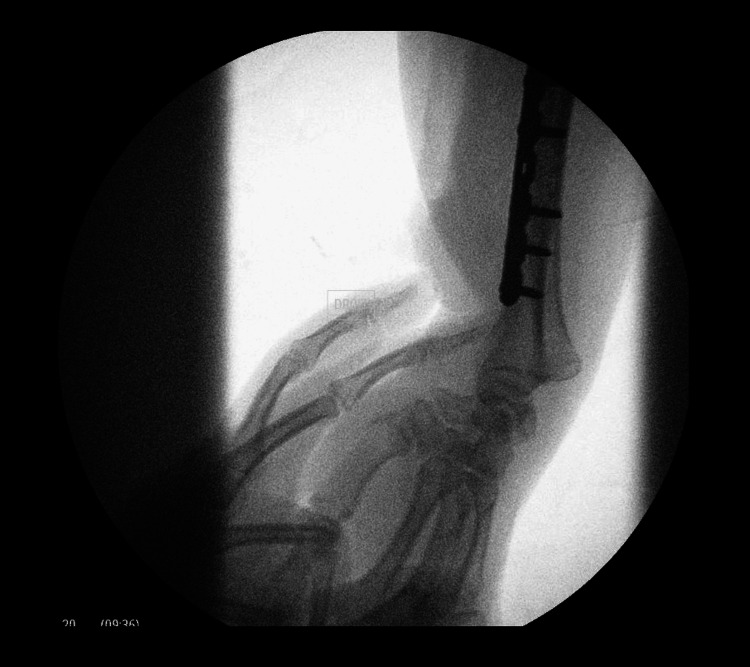
Intraoperative fluoroscopy Fluoroscopy in the operating room showing the instability of the DRUJ in the right wrist. DRUJ: distal radioulnar joint

**Figure 4:**
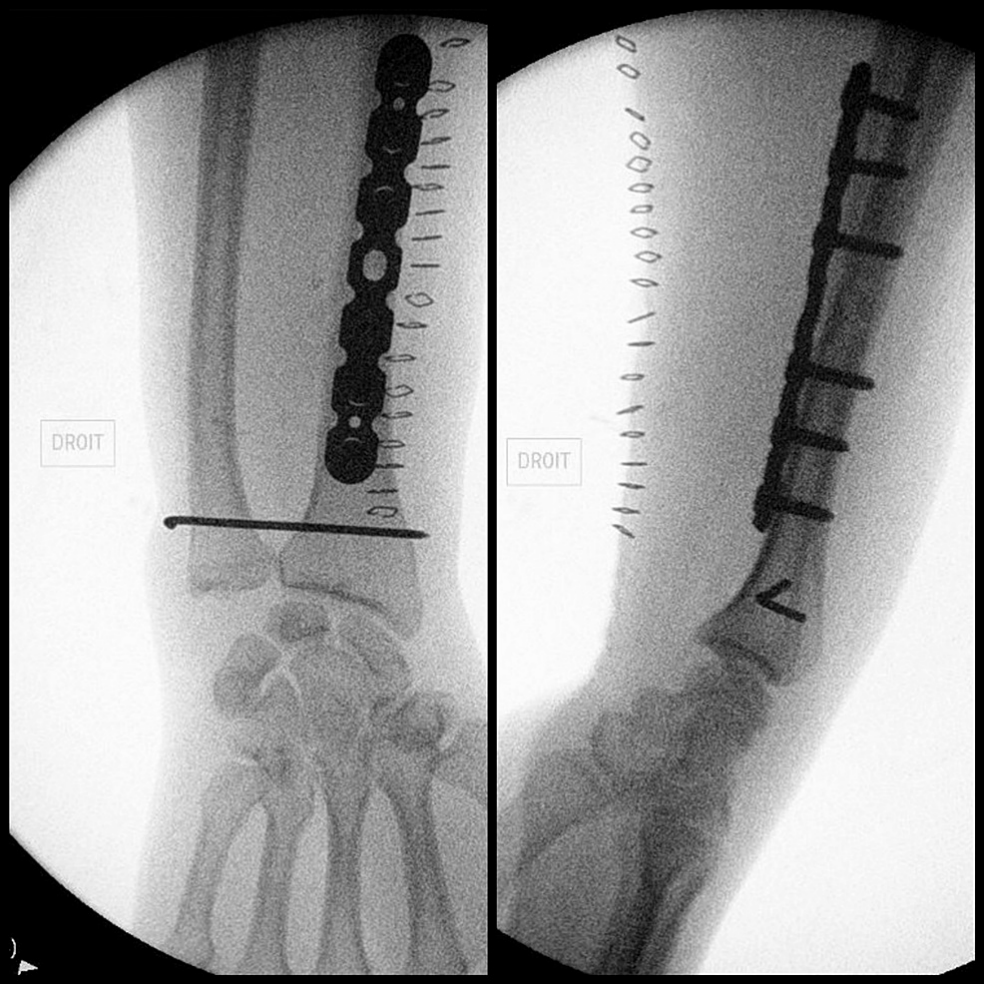
Intraoperative fluoroscopy Fluoroscopic images in the operating room showing final AP (left) and lateral (right) views of the right wrist following DRUJ fixation using an 18-mm pin. DRUJ: distal radioulnar joint

The dynamic testing of the stability of the DRUJ revealed instability, for which DRUJ fixation was performed using an 18-mm pin (Figures 3, 4).

**Figure 5:**
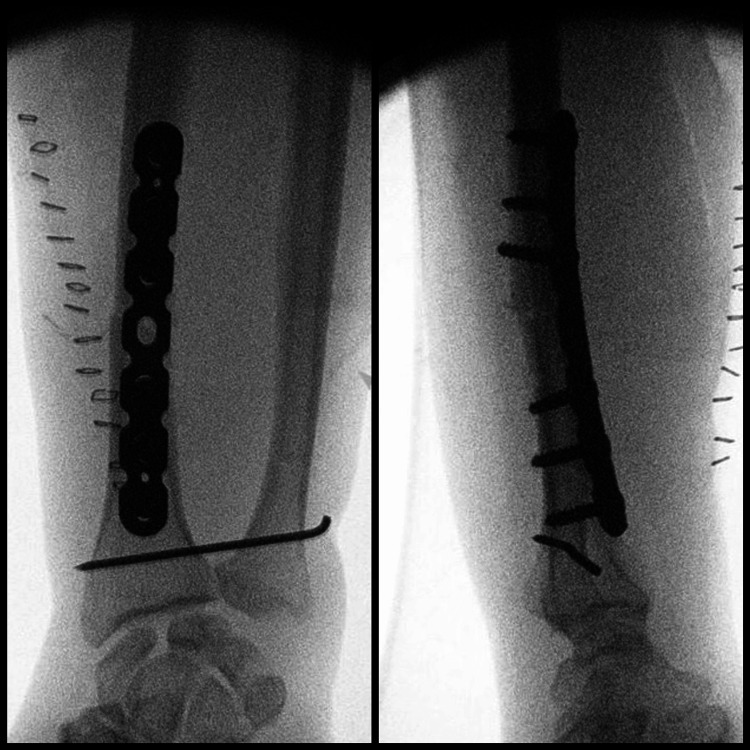
Intraoperative fluoroscopy Fluoroscopic images in the operating room showing final AP (left) and lateral (right) views of the left wrist following DRUJ fixation using an 18-mm pin. DRUJ: distal radioulnar joint

The same procedure was carried out on the left forearm (tourniquet time: 47 minutes) (Figure 5).

Immobilization in a long forearm cast was applied for a period of five weeks, after which the pins fixing the DRUJs were removed in the operating room. The patient underwent several sessions of progressive physiotherapy, beginning with passive range of motion exercises and gradually advancing to active range of motion exercises. As the patient approached a full range of wrist movements, wrist-strengthening exercises were initiated.

**Figure 6:**
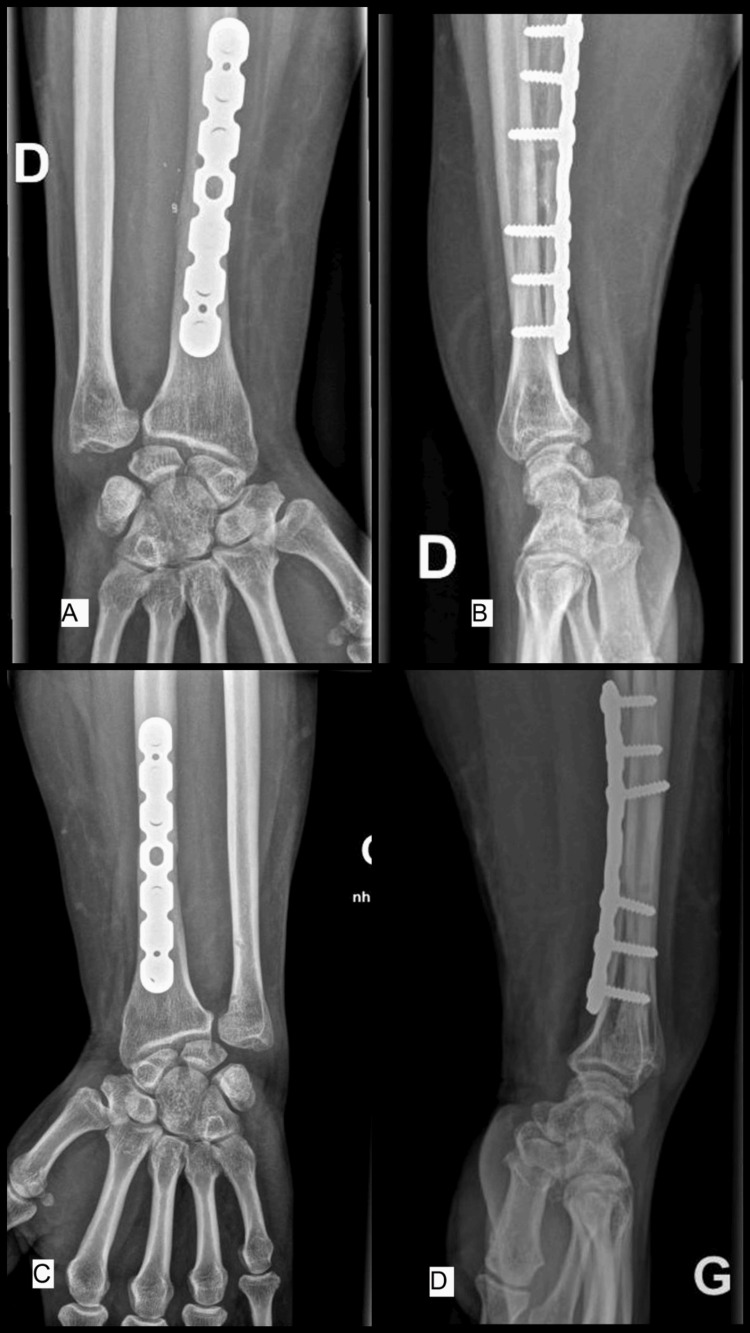
Postoperative X-rays Six-month postoperative AP (A) and lateral (B) views of the right wrist, and AP (C) and lateral (D) views of the left wrist, demonstrating consolidation of the radial fractures and stable DRUJ. DRUJ: distal radioulnar joint

The patient was followed up in the clinic regularly for 16 months. The last radiological follow-up was at six months postoperatively, which revealed bilateral healing of the fracture sites and stability of both DRUJs (Figure 6). At the 16-month postoperative follow-up, the patient was pain-free and had regained full range of motion in both wrists.

## Discussion

Displaced fractures of the distal third of the radius are occasionally accompanied by dislocation of the DRUJ, comprising approximately 7% of forearm fractures. Sir Astley Cooper first described this condition in 1822, and Dr. Riccardo Galeazzi later published his experience with 18 cases in 1934. This type of injury is also known in the literature as a reverse Monteggia fracture [7,8].

Walsh initially classified Galeazzi fractures based on the position of the distal radius and the mechanism of the injury. Type 1 fractures typically occur due to axial force with the wrist extended and the arm maximally supinated, while Type 2 injuries result from axial compression on a pronated forearm. Type II fractures are more frequently observed in children [5].

Historically, Galeazzi maintained reduction of the ulnar head by radially deviating the wrist and stabilizing the radial shaft fracture by pulling on the thumb while the forearm was supinated, using a plaster of Paris cast [10].

Anatomical and rigid fixation of the radius, along with the stability of the DRUJ, are key principles in the treatment regimen typically recommended for adults. This underscores the distinction in managing Galeazzi fractures between adult and pediatric populations. While ORIF is often necessary for adults to achieve favorable outcomes, effective management in children can often be achieved through manipulation under anesthesia and cast immobilization above the elbow. Walsh reported satisfactory results with the nonoperative treatment of pediatric Galeazzi fractures. In contrast, studies have indicated that 80% of adults with Galeazzi fractures experienced poor outcomes when managed non-surgically [5,7].

The stability and adequacy of the DRUJ reduction are evaluated intraoperatively when anatomic reduction and solid fixation of the radius are achieved. The DRUJ is proven unstable if the ulnar head can be moved dorsally out of the sigmoid notch while the forearm is in supination [7,8].

When significant joint instability is noticed, fixation of the ulna and radius with a K-wire after reducing the dislocation is necessary. Joint exploration and ligament repair may be necessary in some cases when DRUJ reduction is difficult. This could be due to the interposition of the extensor carpi ulnaris, extensor digitorum, or extensor minimi tendons, or due to buttonholed bony fragments [8].

For postoperative management, a long arm cast is typically applied for four to six weeks. Most authors recommend immobilizing the forearm in a neutral position.

Early physical therapy and early mobilization sessions are advised to avoid permanent restriction of the range of motion of the wrist and to avoid any delay in returning to work [2].

## Conclusions

Bilateral Galeazzi fracture dislocation is an uncommon and complex injury that carries a high risk of functional disability if not properly managed. Consequently, a detailed clinical assessment and radiological examination are crucial to ensure the diagnosis is not overlooked. Surgical repair is recommended in the adult population. The key to successful surgical treatment is the careful evaluation of DRUJ stability, which, when addressed meticulously, can result in favorable clinical outcomes.
